# Syntheses, characterizations and thermal analyses of four novel unsymmetrical β-diketiminates

**DOI:** 10.1186/2193-1801-2-32

**Published:** 2013-01-31

**Authors:** Siddappa A Patil, Phillip A Medina, Joseph W Ziller, Bradley D Fahlman

**Affiliations:** 1 Department of Chemistry & Science of Advanced Materials Program, Central Michigan University, Mount Pleasant, MI 48859 USA; 2 Department of Chemistry, University of California, Irvine, CA 92697 USA

**Keywords:** Unsymmetrical β-diketiminate, Synthesis, Spectroscopic investigation, X-ray structure, Thermogravimetric analysis

## Abstract

Four novel unsymmetrical β-diketiminates 2-(2,6-diisopropylphenyl)amino-4-(phenyl)imino-2-pentene (**4a**), 2-(2,6-diisopropylphenyl)amino-4-(4-methylphenyl)imino-2-pentene (**4b**), 2-(2,6-diisopropylphenyl)amino-4-(4-methoxyphenyl)imino-2-pentene (**4c**) and 2-(2,6-diisopropylphenyl)amino-4-(4-chlorophenyl)imino-2-pentene (**4d**) were synthesized with a 77-84% yield, and were characterized by spectroscopic methods (^1^H NMR, ^13^C NMR, IR and mass spectrometry), elemental analysis, and X-ray single-crystal diffraction, respectively. Spectroscopic and X-ray single-crystal diffraction analyses determined the structures of the four β-diketiminates. While thermogravimetric analysis (TGA) and differential scanning calorimetry (DSC) showed two distinct endothermic peaks for each β-diketiminate at temperatures of 92.55°C and 221.50°C (**4a**), 93.51°C and 238.82°C (**4b**), 109.60°C and 329.22°C (**4c**), 115.43°C and 243.25°C (**4d**), respectively, corresponding to their melting and boiling points.

## Background

**Scheme 1:**
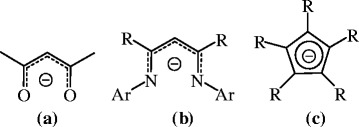
**Schematic diagram of (a) “acac”, (b) “nacnac”, and (c) “cyclopentadienyl” ligands.**

The β-diketiminate class, generally denoted as “nacnac”, or [{ArNC(R)}_2_CH]- (where Ar =aryl and R = Me or another group), occupies a rightful place alongside a narrow list of popular ancillary supports, given its ability to stabilize or generate unique coordination environments and to support reactive organometallic reagents or catalysts (Bourget-Merle et al. 2002; Holland 2008; Mindiola 2006; Cramer and Tolman 2007; Roesky et al. 2004; Piers and Emslie 2002; Rahim et al. 1998). The “nacnac” ligand skeleton is analogous to the “acac” (acetylacetonate) ligand, but the oxygen atoms are exchanged for nitrogen-based moieties such as NR (R = alkyl, silyl, Ar) (Scheme 1). As a result, the substituent at the nitrogen donor atom can allow for steric protection at the metal center unlike “acac” could offer. When small moieties such as H, Me, or the SiMe_3_ on the nitrogen the substance easily forms dimers and allows higher coordination to the metal center, whereas bulky aryl groups on nitrogen usually lead to the isolation of monomeric species with low coordination numbers at the metal.

The first documented cases of β-diketiminate metal complexes were reported by McGeachin (McGeachin 1968), Parks, and Holm (Parks, and Holm 1968) in 1968. The explosion in popularity of “nacnac” amongst synthetic chemists is driven, in part, by the monoanionic nature of the β-diketiminate group, the chelating nature but also variable mode of hapticity, the ease in preparation, and the versatility to tune both electronic and steric parameters. Till to date the N-aryl substituted “nacnac” ligands [HN(Ar)C(Me)CHC(Me)N(Ar)] (Nagendran and Roesky 2008) and [HN(Ar)C(*t*Bu)CHC(*t*Bu)N(Ar)] (Pfirrmann et al. 2009; Ding et al. 2009) (Ar = 2,6-*i*Pr_2_C_6_H_3_) showed to be the best for stabilization of low coordinate metal sites.

The major breakthrough in this area was achieved in the mid 1990’s, when β-diketiminates were used as spectator ligands, thus offering strong metal-ligand bonds like cyclopentadienyls (Scheme 1). In contrast to the latter, β-diketiminates offer a possibility of subtle tuning of their electronic and steric properties by simple variation of the substituents on nitrogen and adjacent carbon atoms.

The availability of straightforward, multigram syntheses for many classes of β-diketiminates has generated widespread popularity of the ligand for coordination and organometallic chemistry. Prototypical symmetrical β-diketimines with N-aryl substituents can be synthesized in one step from commercially available anilines and diones through simple condensation reactions (McGeachin 1968; Stender et al. 2001). β-Diketiminates with aliphatic nitrogen substituents can also be prepared by related condensation routes, but often require harsh reagents such as oxonium salts for complete diimine formation (Kuhn et al. 1999). Other variants of the β-diketiminate scaffold have been recently discussed in a comprehensive review (Bourget-Merle et al. 2002). Herein, we demonstrate a synthetic pathway for unsymmetrical β-diketimines and provide detailed characterization by spectroscopic (^1^H NMR, ^13^C NMR and mass) methods, melting point determination, thermogravimetric analysis (TGA), differential temperature analysis (DTA), and elemental analysis. In addition, the solid state structures of the compounds **4a-d** have been analyzed by single crystal X-ray diffraction.

## Results and discussion

### Synthesis

**Scheme 2:**
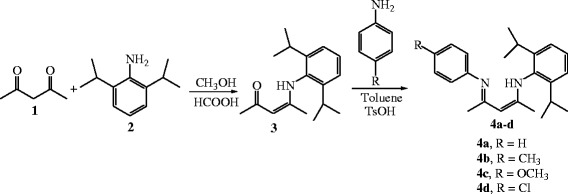
**General reaction scheme for the synthesis of unsymmetrical β-diketiminates 4a-d.**

The synthetic pathway for unsymmetrical β-diketiminates described in this work is outlined in Scheme 2. As depicted in Scheme 2, the unsymmetrical β-diketimines **4a-d** were synthesized in two steps: (1) condensation of acetylacetone with one equiv. of primary aromatic amine 2,6-diisopropylaniline in methanol, with formic acid as catalyst, afforded the (Z)-4-(2,6-diisopropylphenylamino)pent-3-en-2-one intermediate **3**; (2) another equiv. of the other aromatic amine (aniline, 4-methylaniline, 4-methoxyaniline, and 4-chlororlaniline) was pre-treated with *para*-toluenesulfonic acid in a 1 : 1 ratio for 3 h to afford *para*-toluenesulfonate, which was then reacted with the (Z)-4-(2,6-diisopropylphenylamino)pent-3-en-2-one intermediate **3** to give unsymmetrical β-diketimines **4a-d** in good yields. In the second step, the one-pot reaction of (Z)-4-(2,6-diisopropylphenylamino)pent-3-en-2-one **3**, aromatic amine and *para*-toluenesulfonic acid failed to give any target product; the reaction of (Z)-4-(2,6-diisopropylphenylamino)pent-3-en-2-one with the *para*-toluenesulfonic acid took place instead, preventing further reaction with the aromatic amine.

### ^1^H-NMR and ^13^C-NMR spectra

The ^1^H NMR spectra of all the unsymmetrical β-diketimines show a characteristic downfield shift in the range *δ* = 12.87-13.14 ppm for the NH proton and high field shift in the range *δ* = 4.79-4.84 ppm for the methyne proton attributable to the formation of unsymmetrical β-diketimines from (Z)-4-(2,6-diisopropylphenylamino)pent-3-en-2-one and amines. Two sharp singlets observed at the range 1.69-1.83 and 1.56-1.65 ppm are assigned to the protons of the two methyl groups (CH_3_C=NAr and CH_3_CNHAr) of the unsymmetrical β-diketiminates. The resonance due to the four CH_3_ protons (CH(CH_3_)_2_) were observed as a two doublets at the range 1.15-1.19 and 1.09-1.14 ppm while that of CH appeared as a septet at the range 3.12-3.18 ppm.

The ^13^C NMR is in good agreement with the proposed unsymmetrical β-diketiminate structures as well. The ^13^C-NMR spectra of unsymmetrical β-diketiminates showed a peak at the range 161.8-164.1 ppm which is assigned to carbon of the C=N group. The methyne carbon appears at the range 95.9-96.6 ppm. Four peaks at the range 24.3-28.7, 20.3-20.6, 21–22.8 and 20.7-21.2 ppm are due to carbons of the six methyl groups (CH_3_C=NAr, CH_3_CNHAr and CH(CH_3_)_2_) respectively. In addition, their identities have also been confirmed by a molecular ion peak [M^+^] from GC-MS mass spectra.

### FT-IR spectra

The FT-IR spectra for the compounds **4a-d** are recorded in the solid state using the KBr disc technique at the region from 400 to 4000 cm^-1^. The (C=N) bands are observed at 1645–1557 (ν) cm^-1^; the position of these bands varies with the molecular structure, though no regularity can be pointed out. The bands at the range 3055–3072 (ν) cm^-1^ is typical of the NH group. Weak to medium absorptions around 3100–3000 cm^-1^ observed corresponding to the =C–H stretch of aromatic ring.

### X-ray crystal structure

**Figure 1:**
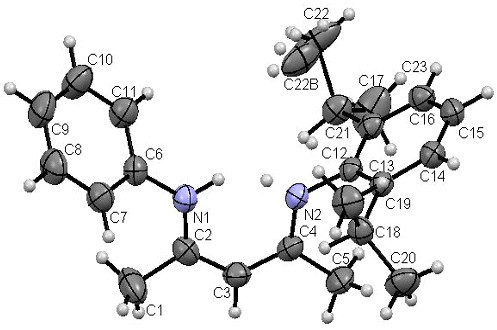
**X-ray diffraction structure of 4a; molecule; thermal ellipsoids are drawn on the 50% probability level.**

**Figure 2:**
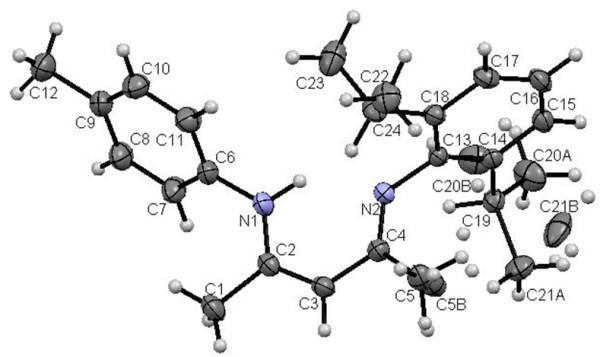
**X-ray diffraction structure of 4b; molecule; thermal ellipsoids are drawn on the 50% probability level.**

**Figure 3:**
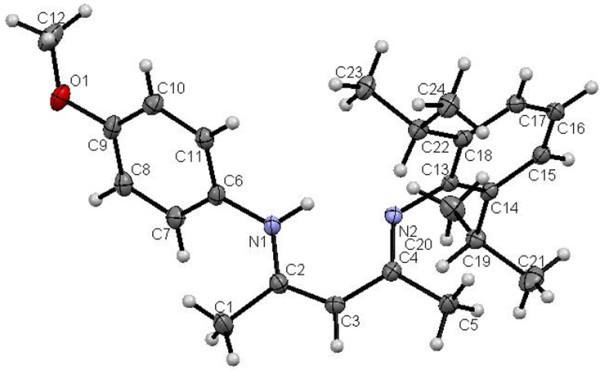
**X-ray diffraction structure of 4c; molecule; thermal ellipsoids are drawn on the 50% probability level.**

**Figure 4:**
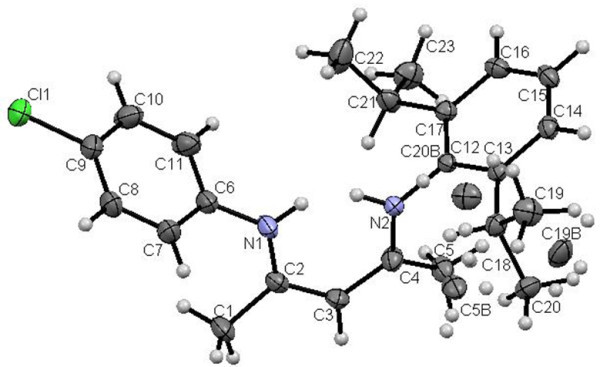
**X-ray diffraction structure of 4d; molecule; thermal ellipsoids are drawn on the 50% probability level.**

**Figure 5:**
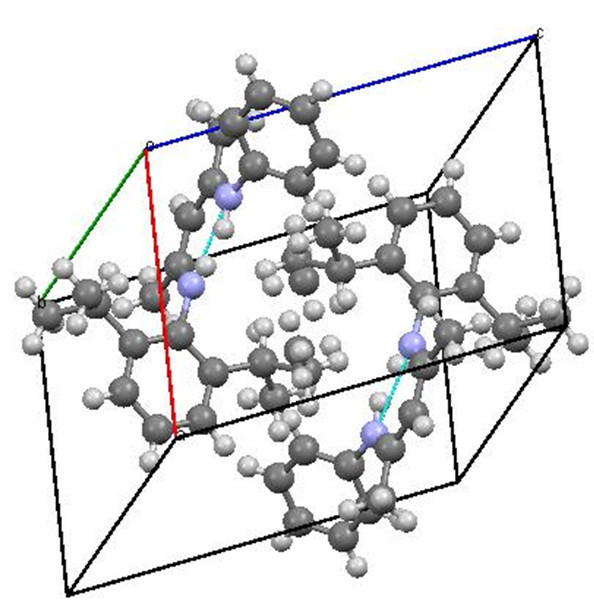
**Perspective view of the molecular packing of 4a showing the intramolecular hydrogen bondings.**

**Figure 6:**
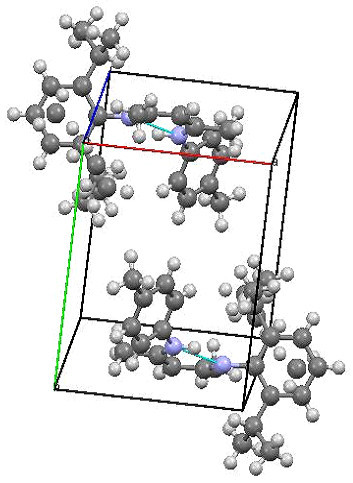
**Perspective view of the molecular packing of 4b showing the intramolecular hydrogen bondings.**

**Figure 7:**
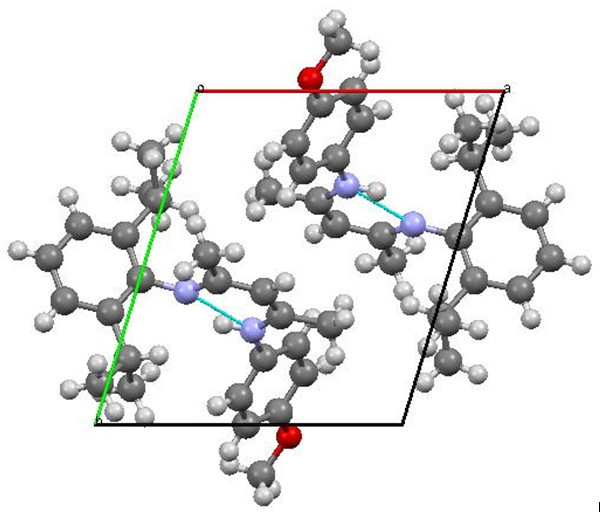
**Perspective view of the molecular packing of 4c showing the intramolecular hydrogen bondings.**

**Figure 8:**
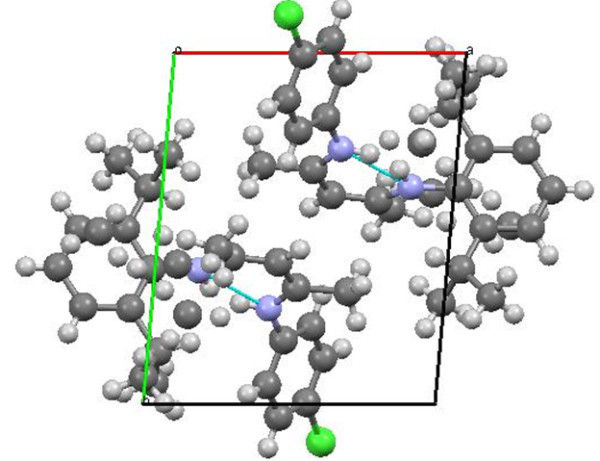
**Perspective view of the molecular packing of 4d showing the intramolecular hydrogen bondings.**

Single crystals of unsymmetrical β-diketiminates **4a-d** were grown by the slow evaporation method using methanol as the solvent at room temperature. The solid state structures of **4a-d** with an atom-numbering scheme are shown in Figures 1, 2, 3 and 4, respectively. The molecular packing diagrams of **4a-d** are displaced in Figures 5, 6, 7 and 8, respectively. A crystallographic data and refinement detail of the compounds **4a-d** is shown in Table 1, whereas selected bond lengths and bond angles are compiled in Table 2. The analysis of the crystal structures of compounds **4a-d** shows that they are coplanar. Compounds **4a-d** have two aromatic rings and a central linkage. Compounds **4a-d** crystallized in the triclinic space group *P* , with two molecules in the unit cell. In these compounds, there is an absence of any lattice held water molecules or organic solvent molecules in the unit cell of the determined structure. The N=C-C=C-NH linkage of the compounds **4a-d** is planar; the bond lengths [Table 2] indicate electron delocalization. The C-C bond distances in aromatic rings are in the normal range of 1.37-1.48 A°, which is characteristic of delocalized aromatic rings. The C-C-C bond angles in aromatic rings are around 120° with the variation being less than 2°, which is characteristic of sp^2^-hybridized carbons. The molecular packing diagrams of all the four unsymmetrical β-diketiminates **4a-d** showed two layers of molecules, which are independently arranged in the unit cell. Molecules forming each layer are not connected through intermolecular hydrogen bonding. In each layer, the molecules are alternatively parallel. The molecular packing diagram also shows the presence of one intra-molecular hydrogen bond. One of the hydrogens, H1 of the NH group, is involved in intra-molecular hydrogen bonding with the N2 of the C=N entity. This hydrogen bonding stabilizes the crystal packing.

**Table 1:** **Crystal data and the structure refinement of the compounds 4a-d**

Identification code	4a	4b	4c	4d
Empirical formula	C_23_ H_30_ N_2_	C_24_ H_32_ N_2_	C_24_ H_32_ N_2_ O	C_23_ H_29_ Cl N_2_
Formula weight	334.49	348.52	364.52	368.93
Temperature (K)	198(2)	143(2)	88(2)	143(2)
Wavelength (Å)	0.71073	0.71073	0.71073	0.71073
Crystal system	Triclinic	Triclinic	Triclinic	Triclinic
Space group	*P*	*P*	*P*	*P*
Unit cell dimensions				
a (Å)	9.6209(4)	8.9868(7)	9.4632(5)	8.8970(6)
b (Å)	11.0221(5)	10.3608(8)	10.3455(5)	10.3481(6)
c (Å)	11.0824(5)	12.0081(9)	12.0086(6)	11.9800(8)
α (^o^)	112.2432(5)	98.8602(9)	92.8431(6)	98.9102(7)
β (^o^)	97.8486(5)	106.9857(8)°	106.1653(5)	107.4484(6)
γ (^o^)	102.1196(5)	93.2382(9)°	105.4519(6)	92.1628(7)
Volume (Å^3^)	1032.76(8)	1050.44(14)	1078.59(9)	1035.36(12)
Z	2	2	2	2
Density (calculated) (Mg/m^3^)	1.076	1.102	1.122	1.183
Absorption coefficient (mm^-1^)	0.062	0.064	0.068	0.193
F_000_	364	380	396	396
Crystal size (mm^3^)	0.42 x 0.33 x 0.26	0.33 x 0.31 x 0.25	0.36 x 0.36 x 0.23	0.38 x 0.37 x 0.26
Theta range for data collection (^o^)	2.04 to 26.37	1.80 to 28.40	1.78 to 28.82	1.81 to 28.54
Index ranges	−12 ≤ *h* ≤ 12, -13 ≤ *k* ≤ 13, -13 ≤ *l* ≤ 13	12 ≤ *h* ≤ 11, -13 ≤ *k* ≤ 13, -15 ≤ *l* ≤ 15	−12 ≤ *h* ≤ 12, -13 ≤ *k* ≤ 13, -15 ≤ *l* ≤ 16	−11 ≤ *h* ≤ 11, -13 ≤ *k* ≤ 13, -15 ≤ *l* ≤ 15
Reflections collected	11311	12098	13056	12115
Independent reflections	4202 [R(int) = 0.0142]	4779 [R(int) = 0.0145]	5172 [R(int) = 0.0138]	4753 [R(int) = 0.0172]
Refinement method	Full-matrix least-squares on F^2^	Full-matrix least-squares on F^2^	Full-matrix least-squares on F^2^	Full-matrix least-squares on F^2^
Completeness to theta = 25.50°	99.7%	99.6%	99.7%	99.6%
Max. and min. Transmission	0.9842 and 0.9742	0.9842 and 0.9794	0.9846 and 0.9760	0.9522 and 0.9295
Data / restraints / parameters	4202 / 0 / 251	4779 / 0 / 277	5172 / 0 / 255	4753 / 0 / 348
Goodness-of-fit on F^2^	1.049	1.037	1.072	1.049
Final R indices [I>2sigma(I)]	R1 = 0.0436, wR2 = 0.1186	R1 = 0.0438, wR2 = 0.1149	R1 = 0.0407, wR2 = 0.1089	R1 = 0.0366, wR2 = 0.0996
R indices (all data)	R1 = 0.0505, wR2 = 0.1248	R1 = 0.0535, wR2 = 0.1225	R1 = 0.0452, wR2 = 0.1126	R1 = 0.0406, wR2 = 0.1031
Largest diff. peak and hole (e.Å^-3^)	0.264 and −0.197	0.264 and −0.201	0.328 and −0.325	0.328 and −0.225

**Table 2:** **Selected bond lengths and bonds angles of the compounds 4a-d**

Bond length (Å)	4a	4b	4c	4d
N(1)-C(2)	1.3269(16)	1.3476(15)	1.3530(13)	1.3293(15)
N(1)-C(6)	1.4151(15)	1.4220(15)	1.4222(12)	1.4148(15)
N(2)-C(4)	1.3231(15)	1.2965(14)	1.3036(13)	1.3154(14)
N(2)-C(12)	1.4270(14)			1.4268(13)
N(2)-C(13)		1.4240(13)	1.4234(12)	
C(1)-C(2)	1.5065(18)	1.5015(16)	1.5039(14)	1.5059(15)
C(2)-C(3)	1.4001(17)	1.3680(16)	1.3777(14)	1.3897(16)
C(3)-C(4)	1.3987(17)	1.4352(16)	1.4382(13)	1.4091(15)
C(4)-C(5)	1.5065(16)	1.551(6)	1.5144(13)	1.561(3)
C(9)-C(12)		1.5093(18)		
O(1)-C(9)			1.3706(12)	
O(1)-C(12)			1.4283(15)	
Cl(1)-C(9)				1.7408(12)
**Bond angles (°)**	**4a**	**4b**	**4c**	**4d**
C(2)-N(1)-C(6)	124.56(11)	126.93(10)	127.61(9)	124.80(10)
C(4)-N(2)-C(12)	123.06(10)			122.54(9)
N(1)-C(2)-C(3)	120.69(11)	120.78(10)	120.80(9)	120.59(10)
N(1)-C(2)-C(1)	121.36(12)	119.51(11)	119.58(9)	121.22(11)
C(3)-C(2)-C(1)	117.90(12)	119.69(11)	119.62(9)	118.17(10)
C(4)-C(3)-C(2)	126.12(11)	126.13(10)	125.84(9)	125.85(10)
N(2)-C(4)-C(3)	121.25(11)	121.18(10)	120.77(9)	121.65(10)
N(2)-C(4)-C(5)	120.14(11)	122.6(2)	122.63(9)	119.30(13)
C(3)-C(4)-C(5)	118.61(11)	114.4(2)	116.60(8)	116.53(13)
C(4)-N(2)-C(13)		120.72(9)	120.70(8)	
C(9)-O(1)-C(12)			116.54(9)	

### Mass spectra and thermal studies

The mass spectra were analyzed by GC-MS. The peaks observed at *m*/*z* 334.49, 348.47, 364.36, and 368.92 suggested the molecular formulas C_23_H_30_N_2_, C_24_H_32_N_2_, C_24_H_32_N_2_O, and C_23_H_29_ClN_2_ of the compounds **4a-d** respectively.

**Figure 9:**
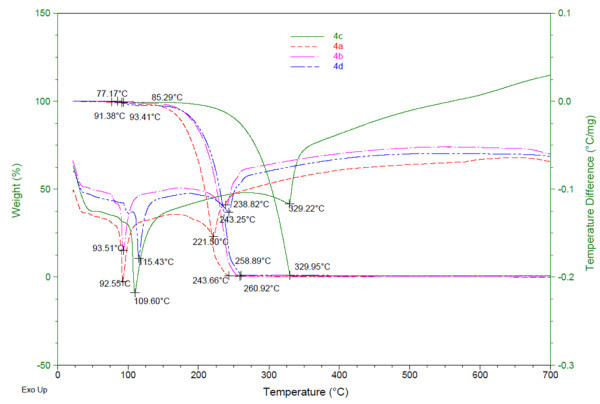
**TGA/DTA of unsymmetrical β-diketiminates 4a-d.**

The thermal behavior of the compounds **4a-d** have been investigated using thermogravimetric techniques in the temperature range from 25°C to 1000°C at a heating rate of 10°C min^-1^ under inert nitrogen gas flow. On the temperature difference curves seen in Figure 9, sharp endothermic peaks were observed at temperatures of 92.55, 93.51, 109.60 and 115.43°C, this indicates that the compounds **4a-d** melt at 92.55, 93.51, 109.60 and 115.43°C, respectively. Further confirmation of their melting point by glass capillary analysis provided values of 93.0, 94.0, 110.0 and 116.0°C for the compounds **4a-d**. The symmetric analogues 2-((phenyl)amino)-4-((phenyl)imino)-2-pentene, 2-((4-methylphenyl)amino)-4-((4-methylphenyl)imino)-2-pentene, 2-((4-methoxyphenyl)amino)-4-((4-methoxyphenyl)imino)-2-pentene and 2-((4-chlorophenyl)amino)-4-((4-chlorophenyl)imino)-2-pentene melt at 51, 73, 94 and 86°C, respectively (Gong and Ma 2008; Tang et al. 2006). At temperatures of 221.50, 238.82, 329.22 and 243.25°C, additional sharp endothermic peaks occurred corresponding to boiling points of the compounds **4a-d**. Onsets of mass loss in the compounds **4a-d** occurred at 77.17, 85.29, 91.38 and 93.41°C and terminated at 243.66, 258.89, 329.95 and 260.92°C as observed by the weight loss curve of the TGA data for each compound.

## Conclusions

All the four unsymmetrical β-diketiminates (**4a-d**) were synthesized. The structures were identified by spectroscopic methods (^1^H NMR, ^13^C NMR, IR and mass), thermogravimetric analysis (TGA), differential temperature analysis (DTA), elemental analysis, and X-ray diffraction analyses. TGA/DTA analyses of all the four unsymmetrical β-diketiminates **4a-d** were studied, showing two distinct endothermic peaks at temperatures of 92.55, 93.51, 109.60, 115.43°C and 221.50, 238.82, 329.22, 243.25°C, corresponding to melting and boiling points, respectively.

### Experimental section

#### General information on reagents and techniques

Reactions were carried out under aerobic conditions. All reagents and solvents are of analytical grade; they were purchased and used without further purification. Acetylacetone, aniline, 4-methylaniline, 4-methoxyaniline, 4-chlororlaniline, formic acid, *para*-toluenesulfoinc acid monohydrate, MgSO_4_, and sodium carbonate were procured commercially from Sigma-Aldrich chemical company, and were used without further purification. Nuclear magnetic resonance (NMR) spectra were obtained using a 1.0% to 2.5% solution in deuterated benzene (C_6_D_6_). ^1^H and ^13^C NMR spectra were recorded on a Varian Mercury 500 MHz spectrometer. Proton and carbon chemical shifts are reported in parts-per-million (δ) with respect to tetramethylsilane (TMS) as internal reference (δ = 0.0 ppm). IR spectra were recorded on a Perkin Elmer Paragon 1000 FT-IR spectrometer employing a KBr disc. Mass spectra were obtained on a GC-MS instrument operating in TOF-MI^+^ mode. CHN analysis was done by Atlantic Microlab using a CE-1108 Elemental Analyzer, and values were within ±0.4% of the theoretical values. Thermogravimetric analyses (TGA) were made with a Pyris TGA instrument. A heating rate of 10^0^C/min was used and samples (5–10 mg) were contained in a platinum pan. The sample compartment was purged with dry nitrogen at 50 mL/min during analysis. TA Thermal Advantage software was used for data analysis. Melting points were determined using a Pyris differential scanning calorimeter (DSC). The crystallographic data for compounds **4a-d** were collected on a Bruker SMART APEX II diffractometer. The APEX2 (Version 2010.9–0, 2010) program package was used to determine the unit-cell parameters and for data collection. The raw frame data was processed using SAINT (Version and 7.68a, 2009) and SADABS Sheldrick (2008a) to yield the reflection data file. Subsequent calculations were carried out using the SHELXTL (Sheldrick 2008b) program. The structures were solved by direct methods and refined on F^2^ by full-matrix least-squares techniques. The analytical scattering factors^21^ for neutral atoms were used throughout the analysis. Hydrogen atoms were included using a riding model.

Crystallographic data for the structures reported in this article have been deposited with the Cambridge Crystallographic Data Center with the deposition numbers 903786, 903787, 903788, and 903789. A copy of the data can be obtained free of charge from the Director, CCDC, 12 Union Road, Cambridge CB2 1EZ, UK [fax: +44 1223 336–033; e-mail: deposit@ccdc.cam.ac.uk or http://www.ccdc.cam.ac.uk].

**4-(2,6-diisopropylphenyl)amino-3-penten-2-one (3)**. 2,6-Diisopropylaniline (1.77 g, 10.0 mmol) was mixed with acetylacetone (1.0 g, 10.0 mmol) in 40 mL of methanol containing a catalytic amount (2 drops) of formic acid. The solution was heated at 85°C for 6–8 h. Removal of volatiles afforded a pale brown oil. This was then stirred with 20 mL 40**–**60°C petroleum ether at −30°C to precipitate a white solid which was filtered off, washed with 2 x 10 mL cold hexane (−78°C), and dried *in vacuo*. Yield: 2.30 g (89%). ^1^H NMR (500 MHz, C_6_D_6_) δ (ppm): 12.66 (1H, s, NH), 7.11-7.06 (m, 1H, ArH), 6.97-6.94 (m, 2H, ArH), 5.08 (s, 1H, CH=C(CH_3_)N), 3.04 (sept, J = 6.8 Hz, 2H, CH(CH_3_)_2_), 2.01 (s, 3H, CH_3_COC), 1.34 (s, 3H, CH_3_CNHAr), 1.02 (d, J = 6.9 Hz, 6H, CH(CH_3_)_2_), 0.96 (d, J = 6.9 Hz, 6H, CH(CH_3_)_2_). ^13^C NMR (100 MHz, C_6_D_6_) δ (ppm): 195.69 (C=O), 162.40 (HC(CNHAr), 146.60 (Ar-C), 134.23 (Ar-C), 128.50 (Ar-C), 123.78 (Ar-C), 95.93 (CH), 29.04 (CH_3_C=O), 28.80 (CH(CH_3_)_2_), 24.53 (CH(CH_3_)_2_, 22.61 (CH(CH_3_)_2_), 18.85 (CH_3_CNHAr). Mass data (TOF MS EI^+^): Calcd for C_17_H_25_NO [M^+^] 259.39, found: 259.21. Anal. Calcd for C_17_H_25_NO: C, 78.72; H, 9.71; N, 5.40; Found: C, 78.61; H, 9.59; N, 5.36. IR (KBr, cm^-1^) ν: 3418 (m), 2972 (w), 16015 (s), 1573 (m), 1515 (m), 1467 (w), 1411 (m), 1397 (m), 1344 (m), 1299 (w), 1203 (w), 1137 (m), 1076 (w), 983 (m), 842 (w), 722 (w), 601 (m), 513 (m), 484 (w).

**2-(2,6-diisopropylphenyl)amino-4-(phenyl)imino-2-pentene (4a).** Aniline (4.55 g, 50.00 mmol), and *para*-toluenesulfonic acid monohydrate (9.510 g, 50.00 mmol) in 100 mL of toluene were stirred for approximately 3 h at room temperature. To the obtained yellow suspension, 4-(2,6-diisopropylphenyl)amino-3-penten-2-one (12.97 g, 50.00 mmol) was added. A Dean-Stark apparatus was attached and the mixture was heated at reflux for 24 h to remove the water. The reaction mixture was cooled to room temperature and all the volatiles were removed under reduced pressure to give a yellow solid. The solid was treated with diethyl ether (100 mL), water (100 mL) and sodium carbonate (10.60 g, 100 mmol), and the obtained mixture was kept stirring. After complete dissolution, the aqueous phase was separated and extracted with diethyl ether. The combined organic phase was dried over MgSO_4_ and rotary evaporated to dryness under reduced pressure to afford a brownish yellow solid. Yellow crystals (12.95 g, 77%) were obtained after recrystallization from methanol. ^1^H NMR (500 MHz, C_6_D_6_) δ (ppm): 13.14 (s, 1H, NH), 7.16-7.14 (m, 3H, ArH), 7.10-7.09 (m, 2H, ArH), 6.97-6.94 (m, 2H, ArH), 6.88-6.85 (m, 1H, ArH), 4.84 (s, 1H, CH=C(CH_3_)N), 3.18 (sept, J = 6.8 Hz, 2H, CH(CH_3_)_2_), 1.82 (s, 3H, CH_3_C=NAr), 1.63 (s, 3H, CH_3_CNHAr), 1.19 (d, J = 7.0 Hz, 6H, CH(CH_3_)_2_), 1.14 (d, J = 7.0 Hz, 6H, CH(CH_3_)_2_). ^13^C NMR (100 MHz, C_6_D_6_) δ (ppm): 163.1 (NCCH_3_), 157.3 (NCCH_3_), 144.9 (Ar-C), 142.8 (Ar-C), 141.1 (Ar-C), 129.2 (Ar-C), 125.2 (Ar-C), 123.4 (Ar-C), 123.3 (Ar-C), 122.7 (Ar-C), 96.6 (CH), 28.7 (CH_3_C=NAr), 24.3 (CH(CH_3_)_2_), 22.7 (CH(CH_3_)_2_), 21.0 (CH(CH_3_)_2_), 20.6 (CH_3_CNHAr). Melting point: 93.0°C. Mass data (TOF MS EI^+^): Calcd for C_23_H_30_N_2_ [M^+^] 334.50, found: 334.49. Anal. Calcd for C_23_H_30_N_2_: C, 82.59; H, 9.04; N, 8.37; Found: C, 82.55; H, 9.01; N, 8.41. IR (KBr, cm^-1^) ν: 3055 (w), 2960 (w), 2920 (w), 2875 (w), 1925 (w), 1873 (w), 1800 (w),1740 (w), 1630 (s), 1547 (s), 1489 (w), 1360 (m), 1275 (m), 1260 (m), 1155 (s), 1100 (w), 1030 (w), 800 (s), 798 (s), 750 (s), 699 (m), 595 (w), 501 (w), 425 (m).

**2-(2,6-diisopropylphenyl)amino-4-(4-methylphenyl)imino-2-pentene (4b).** β-Diketiminate **4b** was synthesized by the same procedure as **4a**. Yield (14.02 g, 80%). ^1^H NMR (500 MHz, C_6_D_6_) δ (ppm): 13.11 (s, 1H, NH), 7.13-7.11 (m, 3H, ArH), 6.89-6.85 (m, 4H, ArH), 4.83 (s, 1H, CH=C(CH_3_)N), 3.16 (sept, J = 6.8 Hz, 2H, CH(CH_3_)_2_), 2.05 (s, 3H, CH_3_),1.83 (s, 3H, CH_3_C=NAr), 1.63 (s, 3H, CH_3_CNHAr), 1.17 (d, 6H, J = 7.0 Hz, CH(CH_3_)_2_), 1.13 (d, J = 7.0 Hz, 6H, CH(CH_3_)_2_). ^13^C NMR (100 MHz, C_6_D_6_) δ (ppm): 163.8 (NCCH_3_), 156.6 (NCCH_3_), 143.7 (Ar-C), 141.6 (Ar-C), 140.5 (Ar-C), 132.8 (Ar-C), 129.8 (Ar-C), 124.8 (Ar-C), 123.4 (Ar-C), 123.1 (Ar-C), 96.4 (CH), 28.7 (CH_3_Ar), 24.3 (CH_3_C=NAr), 22.7 (CH(CH_3_)_2_), 21.1 (CH(CH_3_)_2_), 20.7 (CH(CH_3_)_2_), 20.5 (CH_3_CNHAr). Melting point: 94.0°C Mass data (TOF MS EI^+^): Calcd for C_24_H_32_N_2_ [M^+^] 348.52, found: 348.47. Anal. Calcd for C_24_H_32_N_2_: C, 82.71; H, 9.25; N, 8.04; Found: C, 82.69; H, 9.17; N, 8.03. IR (KBr, cm^-1^) ν: 3149 (w), 3072 (w), 3015 (w), 2960 (m), 1635 (w), 1598 (s), 1542 (m), 1468 (m), 1439 (m), 1388 (w), 1255 (m), 1236 (s), 1180 (w), 1120 (m), 1105 (m), 1037 (m), 760 (m), 741(m), 664 (w), 615 (w), 602 (w), 557 (w).

**2-(2,6-diisopropylphenyl)amino-4-(4-methoxyphenyl)imino-2-pentene (4c).** β-Diketiminate **4c** was synthesized by the same procedure as **4a**. Yield (15.25 g, 84%). ^1^H NMR (500 MHz, C_6_D_6_) δ (ppm): 13.04 (s, 1H, NH), 7.14-7.11 (m, 3H, ArH), 6.87-6.84 (m, 2H, ArH), 6.65-6.63 (m, 2H, ArH), 4.84 (s, 1H, CH=C(CH_3_)N), 3.26 (s, 3H, OCH_3_), 3.17 (sept, J = 6.8 Hz, 2H, CH(CH_3_)_2_), 1.81 (s, 3H, CH_3_C=NAr), 1.65 (s, 3H, CH_3_CNHAr), 1.19 (d, J = 7.0 Hz, 6H, CH(CH_3_)_2_), 1.14 (d, J = 7.0 Hz, 6H, CH(CH_3_)_2_). ^13^C NMR (100 MHz, C_6_D_6_) δ (ppm): 164.1 (NCCH_3_), 156.8 (NCCH_3_), 156.6 (Ar-C), 144.2 (Ar-C), 140.2 (Ar-C), 136.6 (Ar-C), 125.0 (Ar-C), 124.6 (Ar-C), 123.4 (Ar-C), 114.5 (Ar-C), 95.9 (CH), 54.9 (OCH_3_Ar), 28.7 (CH_3_C=NAr), 24.2 (CH(CH_3_)_2_), 22.8 (CH(CH_3_)_2_), 21.2 (CH(CH_3_)_2_), 20.3 (CH_3_CNHAr). Melting point: 110.0°C. Mass data (TOF MS EI^+^): Calcd for C_24_H_32_N_2_O [M^+^] 364.52, found: 364.36. Anal. Calcd for C_24_H_32_N_2_O: C, 79.08; H, 8.85; N, 7.68; Found: C, 79.10; H, 8.72; N, 7.57. IR (KBr, cm^-1^) ν: 3057 (m), 2971 (m), 2925 (w), 2878 (m), 2832 (w), 1631 (m), 1548 (m), 1520 (s), 1472 (s), 1400 (s), 1320 (m), 1289 (m), 1260 (w), 1251 (m), 1233 (w), 1169 (m), 1115 (m), 930 (w), 795 (m), 759 (m), 753 (w), 740 (m), 679 (w), 641 (w), 597 (m), 578 (w), 415 (m).

**2-(2,6-diisopropylphenyl)amino-4-(4-methoxyphenyl)imino-2-pentene (4c).** β-Diketiminate **4d** was synthesized by the same procedure as **4a**. Yield (15.10 g, 82%). ^1^H NMR (500 MHz, C_6_D_6_) δ (ppm): 12.87 (s, 1H, NH), 7.14-7.08 (m, 3H, ArH), 7.00-6.98 (m, 2H, ArH), 6.63-6.60 (m, 2H, ArH), 4.79 (s, 1H, CH=C(CH_3_)N), 3.12 (sept, J = 6.8 Hz, 2H, CH(CH_3_)_2_), 1.69 (s, 3H, CH_3_C=NAr), 1.56 (s, 3H, CH_3_CNHAr), 1.15 (d, J = 7.0 Hz, 6H, CH(CH_3_)_2_), 1.09 (d, J = 7.0 Hz, 6H, CH(CH_3_)_2_). ^13^C NMR (100 MHz, C_6_D_6_) δ (ppm): 161.8 (NCCH_3_), 159.2 (NCCH_3_), 144.9 (Ar-C), 142.2 (Ar-C), 141.0 (Ar-C), 129.2 (Ar-C), 128.3 (Ar-C), 125.9 (Ar-C), 123.6 (Ar-C), 123.5 (Ar-C), 96.6 (CH), 28.7 (CH_3_C=NAr), 24.4 (CH(CH_3_)_2_), 22.7 (CH(CH_3_)_2_), 20.7 (CH(CH_3_)_2_), 20.6 (CH_3_CNHAr). Melting point: 116.0°C. Mass data (TOF MS EI^+^): Calcd for C_23_H_29_ClN_2_ [M^+^] 368.94, found: 368.92. Anal. Calcd for C_23_H_29_ClN_2_: C, 74.88; H, 7.92; Cl, 9.61; N, 7.59; Found: C, 74.89; H, 7.90; Cl, 9.58; N, 7.55. IR (KBr, cm^-1^) ν: 3062 (s), 2960 (m), 1670 (s), 1558 (m), 1530 (w), 1495 (m), 1450 (m), 1417 (s), 1325 (m), 1271 (w), 1239 (m), 1215 (w), 1182 (m), 1120 (m), 1051 (m), 1030 (m), 930 (w), 864 (m), 837 (w), 800 (w), 745 (m), 677 (w), 635 (w).

## References

[CR1_151] *APEX2 Version 2010.9–0*. Madison, WI: Bruker AXS, Inc; 2010.

[CR2_151] Bourget-Merle L Lappert MF Severn JR The chemistry of β-diketiminatometal complexes Chem Rev 2002 102 3031 3066 10.1021/cr010424r 12222981

[CR3_151] Cramer CJ Tolman WB Mononuclear Cu–O_2_ complexes: geometries, spectroscopic properties, electronic structures, and reactivity Acc Chem Res 2007 40 601 608 10.1021/ar700008c 17458929 PMC2593863

[CR4_151] Ding K Pierpont AW Brennessel WW Lukat-Rodgers G Rodgers KR Cundari TR Bill E Holland PL Cobalt-dinitrogen complexes with weakened N-N bonds J Am Chem Soc 2009 131 9471 9472 10.1021/ja808783u 19537787 PMC2746749

[CR5_151] Gong S Ma H (2008) β-Diketiminate aluminium complexes: synthesis, characterization and ring-opening polymerization of cyclic esters Dalton Trans 2008 0 3345 3357 10.1039/b802638f 18560667

[CR6_151] Holland PL Electronic structure and reactivity of three-coordinate iron complexes Acc Chem Res 2008 41 905 914 10.1021/ar700267b 18646779 PMC2587011

[CR7_151] Kuhn N Fahl J Fuchs S Steimann M Henkel G Maulitz AHZ Vinamidin-chelate des aluminiums und Ggalliums Z Anorg Allg Chem 1999 625 12 2108 2114 10.1002/(SICI)1521-3749(199912)625:12<2108::AID-ZAAC2108>3.0.CO;2-K

[CR8_151] McGeachin SG Synthesis and properties of some β-diketimines derived from acetylacetone, and their metal complexes Can J Chem 1968 46 1903 1912 10.1139/v68-315

[CR9_151] Mindiola DJ Oxidatively induced abstraction reactions. A synthetic approach to low-coordinate and reactive early transition metal complexes containing metal-ligand multiple bonds Acc Chem Res 2006 39 813 821 10.1021/ar0500113 17115721

[CR10_151] Nagendran S Roesky HW The Chemistry of aluminum(I), silicon(II), and germanium(II) Organometallics 2008 27 457 492 10.1021/om7007869

[CR11_151] Parks JE Holm RH Synthesis, solution stereochemistry, and electron delocalization properties of bis(.beta.-iminoamino)nickel(II) complexes Inorg Chem 1968 7 1408 1416 10.1021/ic50065a029

[CR12_151] Pfirrmann S Limberg C Herwig C Stöβer R Ziemer B A Dinuclear nickel(I) dinitrogen complex and its reduction in single-electron steps Angew Chem Int Ed 2009 48 3357 3361 10.1002/anie.200805862 19322853

[CR13_151] Piers WE Emslie DJH Non-cyclopentadienyl ancillaries in organogroup 3 metal chemistry: a fine balance in ligand design Coord Chem Rev 2002 233–234 131 155 10.1016/S0010-8545(02)00016-4

[CR14_151] Rahim M Taylor NJ Xin S Collins S Synthesis and structure of acyclic bis(ketenimine) complexes of zirconium Organometallics 1998 17 1315 1323 10.1021/om970862h

[CR15_151] Roesky HW Singh S Jancik V Chandrasekhar V A Paradigm change in assembling OH functionalities on metal centers Acc Chem Res 2004 37 969 981 10.1021/ar0402154 15609989

[CR16_151] *SAINT Version 7.68a*. Madison, WI: Bruker AXS; 2009.

[CR17_151] Sheldrick GM SADABS, Version 2008/1 2008 Inc, Madison, WI Bruker AXS

[CR18_151] Sheldrick GM SHELXTL, Version 2008/4 2008 Inc, Madison, WI Bruker AXS

[CR19_151] Stender M Wright RJ Eichler BE Prust J Olmstead MM Roesky HW Power PP The synthesis and structure of lithium derivatives of the sterically encumbered β-diketiminate ligand [{(2,6-Pr^i^_2_H_3_C_6_)N(CH_3_)C}_2_CH]^–^, and a modified synthesis of the aminoimine precursor Dalton Trans 2001 0 3465 3469

[CR20_151] Tang LM Duan YQ Li XF Li YS Syntheses, structure and ethylene polymerization behaviour of β-diiminato titanium complexes J Organomet Chem 2006 691 2023 2030 10.1016/j.jorganchem.2005.12.053

